# Identifying domains of health-related quality of life: the perspective of individuals with low back pain

**DOI:** 10.1186/s41687-023-00597-5

**Published:** 2023-07-26

**Authors:** O Eilayyan, A Gogovor, D Zidarov, N Mayo, S Ahmed

**Affiliations:** 1 School of Physical & Occupational Therapy, Faculty of Medicine, McGill University, 3654 Prom Sir-William-Osler, Montréal, QC H3G 1Y5 Canada; 2 Centre for Outcomes Research and Evaluation (CORE), Division of Clinical Epidemiology, McGill University Health Center Research Institute, McGill University, Montréal, QC Canada; 3 Centre de recherche interdisciplinaire en réadaptation (CRIR), Constance Lethbridge Rehabilitation Center, Montréal, QC Canada; 4 Division of Experimental Medicine, Faculty of Medicine, McGill University, Montréal, QC Canada; 5 Faculté de Médecine, École de réadaptation, Université de Montréal, Montréal, QC Canada; 6 Institut universitaire sur la réadaptation en déficience physique de Montréal, Centre intégré universitaire de santé et de services sociaux du Centre-Sud-de-l’Île-de-Montréal, Montréal, QC Canada

**Keywords:** Low back Pain, Health-related quality of life, Patient-reported outcomes

## Abstract

**Background:**

Identifying the most relevant HRQOL domains for LBP from the perspective of individuals with lived experience with LBP is necessary to prioritize domains that will be most informative for evaluating the impact of pain and interventions while overcoming the burden of using long-form assessment tools. This study aimed to identify which domains of HRQOL are most important from the perspective of individuals with chronic LBP.

**Methods:**

Semi-structured interviews were conducted with 26 individuals with LBP. Participants first responded to questions related to the impact of their LBP on their HRQOL. Then, using a card sorting method, they were asked to select and indicate HRQOL domains that were most relevant to them from a list of 18 cards that represented different HRQOL domains. Participants were asked to explain the reasoning for their selection.

**Results:**

Participants identified physical activity restriction (50%), severity of pain (31%), social activity restriction (23%), and work performance restriction (23%) as the most important domains. The most frequently selected HRQOL domains during card sorting were social function (69%), pain intensity (62%), physical function (58%), fatigue (58%), and pain interference (42%).

**Conclusion:**

The most important domains of HRQOL perceived by participants were pain intensity, social function, physical function, fatigue, and pain interference. Identifying these domains will inform clinical decision-making and guide treatment choices for health care providers.

**Supplementary Information:**

The online version contains supplementary material available at 10.1186/s41687-023-00597-5.

## Introduction

Low back pain (LBP) is a common condition worldwide; approximately 50–80% of adults suffer from LBP at least once during their lives [1, 2]. LBP represents a significant health problem because of its consequences for physical and emotional health, employment, personal costs of disability, and societal costs of providing care [3–6]. The annual direct cost of care for LBP in Canada has been estimated in billions of dollars ($6 to $12 billion) [7].

Improving individuals’ health-related quality of life (HRQOL), including pain intensity reduction and increased participation in everyday activities, is the ultimate goal of rehabilitation interventions for LBP [8, 9]. It is also considered an important outcome in clinical studies [10–13]. HRQOL is a term referring to “the health aspects of quality of life, generally considered to reflect the impact of disease and treatment on disability and daily functioning. HRQOL also reflects the impact of perceived health on an individual’s ability to live a fulfilling life” [14].

Consequently, there is growing interest among clinicians in monitoring HRQOL in clinical practice to guide clinical decision-making, including treatment planning and referral to appropriate services [15]. Implementing the routine use of a standardized HRQOL measure in clinical practice could improve the quality of care provided to patients [16]. However, HRQOL is a subjective concept [17], and the relevant domains may vary from one patient to another. This makes it challenging for clinicians to identify the right questions to ask individuals in an efficient manner. The literature shows that there is inconsistency among the domains of LBP measured in research and clinical practice [18].

Identifying the most relevant HRQOL domains of LBP from the perspective of both clinicians and patients is necessary to develop a more specific measure to help assess individuals with LBP. Recently, different initiatives have identified the important domains of chronic pain in general [19] or disease-specific chronic pain conditions (e.g., nonspecific LBP) [20, 21]. For LBP, the key HRQOL domains to be evaluated in both clinical practice and research were identified by the National Institute of Health (NIH) [22] according to clinicians’ and experts’ perceptions, mainly for clinical trials. The key domains that were identified by NIH experts included “pain intensity, pain interference, physical function, depression, sleep disturbance, and catastrophizing” [22]. However, the NIH initiative did not include patients’ perspectives in the process of key HRQOL domain selection.

To our knowledge, there are no recommendations for HRQOL domains for LBP that should be systematically collected in clinical practice, are perceived to be relevant by individuals with LBP and are developed specifically for individuals treated in settings that employ multimodal and multidisciplinary approaches. Our team evaluated the most important domains perceived as important for individuals with nonspecific chronic pain. Whether these are the same for individuals with LBP is not known [23]. Identifying the most important domains as perceived by individuals with LBP may improve the quality of care by measuring them as part of usual clinical care to inform clinical decision-making and guide intervention choices for multidisciplinary care. It may also be valuable for secondary use in comparative effectiveness research and quality improvement initiatives. This study is a first step toward implementing the collection of HRQOL measures in LBP care. Furthermore, selecting specific domains that are important to individuals with LBP to consistently measure in clinical care and adding additional domains only when relevant for an individual patient decreases the burden on both patients and clinicians. The objective of this study was to identify which domains of HRQOL are most important to evaluate in clinical practice from the perspective of individuals with chronic LBP.

## Methods

### Study design

This study adopted a triangulation mixed-method design [24] in which quantitative and qualitative data were used to evaluate individuals’ perception of the impact of LBP on HRQOL (qualitative part) followed by a card sorting methodology to identify the most important domains of HRQOL (quantitative part).

### Population

The participants were individuals living with non-specific LBP recruited from four Health and Social Services Centers in Québec, where they received interdisciplinary intervention provided by physicians, nurses, physiotherapists, and psychologists. Individuals who had non-specific LBP for at least 3 months and were proficient in French or English were included.

### Ethical approval

#### Ethics approval

was obtained from the Ethics Review Board of the Centre for Interdisciplinary Research in Rehabilitation of Greater Montréal (CRIR) (MP-CUSM-12-220 GEN), and written informed consent was obtained from all participants.

### Study procedure

Semi-structured interviews were used to identify the most important domains of HRQOL as perceived by individuals with LBP. The Patient-Reported Outcomes Measurement Information System (PROMIS) framework was used to guide the interview questions [25]. PROMIS is an initiative of the NIH that uses the WHO definition of health, which is described as physical, mental, and social health [26, 27]. Therefore, the PROMIS framework divides HRQOL domains into three main categories: physical, mental, and social health. Each category includes different domains, such as physical function, pain intensity, and depression. In addition, the PROMIS includes various patient-centered measures that assess different domains of health. The PROMIS framework was used in this study because it is a measurement framework rather than a health system framework that helps in developing measures to assess patients’ experience of their own health [28]. It also specifies the subcategories of health domains (e.g., pain, physical function, depression).

Eighteen of the PROMIS domains were presented to participants during the interviews. Not all PROMIS domains were used in the interviews because some of them are not relevant to LBP, such as domains related to the gastrointestinal system.

The interview guide was adapted from Paap et al. (2014) [29] and consisted of two parts: an open-ended question and a card sorting task. First, each participant was asked, *“How does LBP affect your quality of life?”* to assist in thinking about the most important domains. Participants were then shown 18 cards with the PROMIS HRQOL domains accompanied by randomly selected example items and descriptions of the domains. These two domains were combined because participants had difficulty differentiating between them. The merged domain was used in the results and discussion of this study. Participants were invited to choose and rank five domains from all the domains that they considered most affected and important for them with regard to LBP. Research shows that selecting and ranking five domains is feasible [29–32]. Participants were also asked to explain the reasoning for their selection and ranking. Appendix 1 and 2 present the interview guide [29] and the 18 domains of PROMIS HRQOL [33]. At the end of the study, social function was merged from two PROMIS domains: “*ability to participate in social roles and activities*” and “*satisfaction with social roles and activities*”.

Four members of the research team conducted the interviews and attended a 2-hour training session provided by the first author (OE) to standardize the interview process. Interviews took place at the clinic where each participant received treatment for LBP, and they were audio recorded and transcribed. Each interview lasted approximately 30 min.

### Data analyses

We conducted a deductive thematic analysis (**qualitative part**) [34, 35] of the interviews by coding and assigning the codes to the PROMIS framework domains [33]. The participants’ statements were coded and interpreted. In addition, the number of times each domain was selected from the 18 cards was counted (**quantitative part**). The card-sorting part was used to identify and select the most important HRQOL domains. Two independent reviewers (OE and AG), who were PhD students trained in both qualitative and quantitative analysis and with previous qualitative analysis experience, coded the participants’ statements to increase the reliability of the coding procedure. In cases of uncertainty, SA was involved as a third reviewer to reach a final consensus. Data obtained from the first part (open-ended question) and the second part (card sorting task) were analyzed separately, but the same coding procedure was used. For the open-ended question, similar participant statements were divided into units of meaning, which could be part of a sentence or several sentences. The units were then interpreted and coded according to the PROMIS domain framework (subthemes such as pain and depression), and similar subthemes referred to the PROMIS health categories (i.e., physical, mental or social health). For example, pain, physical function and fatigue coded to physical health, while depression and anxiety coded to mental health.

Data from both the open-ended question and card sorting were triangulated. Card sorting was used to select the most important domains by counting the number of domains chosen by participants (i.e. out of 26 participants 8 chose domain x) and by thematically analyzing the reasons for the selections (to understand how the selected domains were affected by LBP). The open-ended question was not used in this study for the domain selection process, but it was used to understand how LBP affected the selected HRQOL domains.

### Sample size

For practical reasons, we used the same sample size for both parts, the card sorting and the open-ended question. This approach was adopted previously by Paap et al. [29]. The literature suggests a sample size of 5 to 50 participants as adequate to conduct interviews [36]. Patient interviews were stopped when saturation of the data was reached. To assess data saturation, we analyzed the interview data of 13 consecutive patients in each group (i.e., English and French interviews). No new themes emerged after the 10th interview in each group. Twenty-six participants were interviewed to identify the most important HRQOL domains in the context of LBP.

## Results

Among the 26 participants who were interviewed in this study, thirteen were English-speaking and thirteen were French-speaking. 46% of participants were females, and the average age was 54 years (SD = 14.7). On average, the participants had LBP for 7 ± 9 years. 62% of the participants had comorbidities in addition to LBP; most of these comorbidities were related to musculoskeletal problems.

The output of the open-ended interview question and of the HRQOL card sorting are presented separately in the following result sections accompanied by quotations drawn from the participants’ statements. Tables 1 and 2 present examples of coding for a few open-ended question statements and for the pain intensity domain statements (card sorting), respectively.

**Table 1:** Examples of codes and their interpretation from the open-ended question

Main theme	Subtheme	Selected unit	Interpretation
Physical Health	Pain Description	“First of all, it’s painful. My legs have pain, not so much my back. I have a little bit of neuropathy, more on the right side than left side so I am taking medications cause insomnia”	The pain is severe, and medication is needed to decrease it
Physical Health	Restriction in activities	“Sometimes pain affects my ability to performing tasks as well as I want to, and sometimes it affects my. I guess my willingness to want to do some activities”.	Limitation in performing tasks and activities
Social Health	Restriction in social activity	*“*Sometimes I notice it impacts my behaviors, and I am less active, and it would definitely affect my personal relationship sometimes*”.*	Interference in personal/social life
Social Health	Restriction in work performance	“I work part time now, I don’t work full time. I am a hair dresser and its too hard on the back. So I am on my feet and you are bending all over when you do the customer’s hair, so I cut my work in half now”	Reduced work hours because of LBP
Mental Health	Anxiety	“Some days if I am in pain, I might have more redundancy to feel a little bit more like anxious or helpless or something like that”	Feeling anxious because of pain

**Table 2:** Examples of codes and their interpretation for the pain intensity domain from card sorting

Theme	Subtheme	Selected unit	Interpretation
Description	Severe pain	Pain intensity, because sometimes is better than other days. Mostly I have bad days. So I would say pain intensity could be so bad	Pain is severe and constant
Determinant	Being in the same position for a long time	So this is the pain you know, it reduces and sit and stand for long time (anything I do for a little bit long) causes pain again	Not changing body position exacerbates pain
Coping with pain	Change in body position decreases the pain	*Oups… oh yo yoye que ça fait mal. Mais là, c’est pas si pire, tu te repositionnes*. [Oups… oh yo yoye that hurts. But there, it’s not so bad, you reposition yourself]	Repositioning to reduce pain
Effect/Consequence	Restriction in activities	I used to dance, I used to walk. I cannot do that, I walk slower that what I used to do and forget dancing, not happening.	Limitation in activities
Autonomy	Autonomy	*Pis même que des fois… j’ai besoin d’aide finalement quand… mes pires jours de crise.* [Even that sometimes… I finally need help when… my worst days of crisis.]	Need help

### Open-ended question: “How does LBP affect your quality of life?”

In total, participants made seventy-two statements regarding the effect of LBP on their quality of life. These statements were mapped to one of the main themes, physical, mental, or social health. The PROMIS framework was used to guide the analysis [37]. Statements from the thematic analysis were classified into subthemes. Thirty-five statements were mapped to physical health, producing seven subthemes; nineteen statements were mapped to social health, producing five subthemes; and seventeen statements were mapped to mental health, producing ten subthemes. Finally, one statement was mapped to general health. The most frequent subthemes, described below, were *general physical activity restriction (N = 22, 31%)*, *work performance restriction (N = 7, 10%), social activity restriction (N = 6, 8%)*, and *pain description (severe and constant pain) (N = 5, 7%)*. Table 3 presents the main themes and subthemes that emerged from the open-ended question statements.

**Table 3:** The main themes and subthemes from the open-ended question

Main themes (number of occurrences)	Subthemes (number of occurrences)
Physical Health (35)	Physical activity restriction (general, specific, and daily activities and sport) (22)
	Severe pain (5)
	Constant pain (3)
	Pain exacerbation/alleviation factors (2)
	Fatigue (2)
	Sleep disturbance (2)
	Coping with pain (1)
Social Health (19)	Restriction in work performance (7)
	Restriction in social activity (6)
	Loss of leisure (3)
	Restriction in personal/family relationship (2)
	Social isolation (1)
Mental Health (17)	Anxiety (3)
	Main concern in life (3)
	Mood changes (2)
	Anger (2)
	Depression (2)
	Coping with emotional impairment (1)
	Lack of concentration (1)
	Less enthusiasm (1)
	Stress (1)
	Bother (1)
General Health (1)	General Health (1)

#### Pain description

Eight participants indicated that pain was a problem as a result of their LBP condition. It was described as severe and constant pain; it never entirely went away. However, one participant stated that she tried to live with the pain and not let it affect her life.

*“I am in a little bit more pain than usual” P7*

“*Y a toujours… la douleur disparait jamais complètement. Y a toujours une douleur qui est présente.* [*There is always.. the pain never completely disappears. There’s always a pain that is present].” P19*

*“I do not let it to stop me from what I want to do. So I think it does not affect my quality of life.” P1*

#### Physical activity restriction

Restriction in activities was the most frequent and common subtheme that emerged from the open-ended question, and it was mapped to physical health. Twenty-two statements were made by thirteen participants. Participants mainly referred to restriction in terms of physical and general tasks and daily life activities, including housework, bathing, dressing, walking, and running. Most of the participants in the study indicated that they had restrictions in general physical activities (daily and physical activities) and they did not perform these activities as they did before having LBP.

*“It is hard to walk, forget running, forget dancing. I cannot sit for long periods of time, I have to get up.” P12*

*“When I go shopping or go to buy some stuff, I go one by one to go upstairs because I live on the third floor. I have a little far to walk at least 10 mins so carry two bags at the time not like five bags. I used to lift all bags in one shot and now I cannot do that” P16*

*“I love to play hockey, but I cannot play hockey, activities around the house. I have to stop often to release pain from my back, but it depends how you feel sometimes” P17*

#### Social activity restriction

Six participants indicated that they had restrictions in social activities, including participation in family activities and communication with other people. One participant stated that she isolated herself socially because of LBP. In total, there were 7 statements related to social activity, including social isolation.

*“Sometimes, I notice it impacts my behaviors and I am less active, and it would definitely affect my personal relationships sometimes”. P7*

*“Ça peut m’empêcher de faire certaines activités et de rencontrer les gens et de sortir ou d’aller les accueillir ou d’aller faire n’importe quoi… prendre une marche, aller au musée… n’importe quoi… à sortir. [It can prevent me from doing certain activities and meeting people and going out or receiving people or doing anything, taking a walk, going to the museum... anything... to go out].” P3*

#### Work performance restriction

Six participants made seven statements indicating that LBP affected and interfered with their work performance. The participants stated that they could not work or they needed to reduce their work hours.

*“I work part time now. I do not work full time. I am a hairdresser, and it’s too hard on the back”. P21*

*“Si je veux travailler, travailler physique, je travaille pas longtemps. [If I want to work, physical work, I don’t work for long time]”. P11*

### Selecting the most relevant PROMIS HRQOL domains – card sorting

**Fig. 1:**
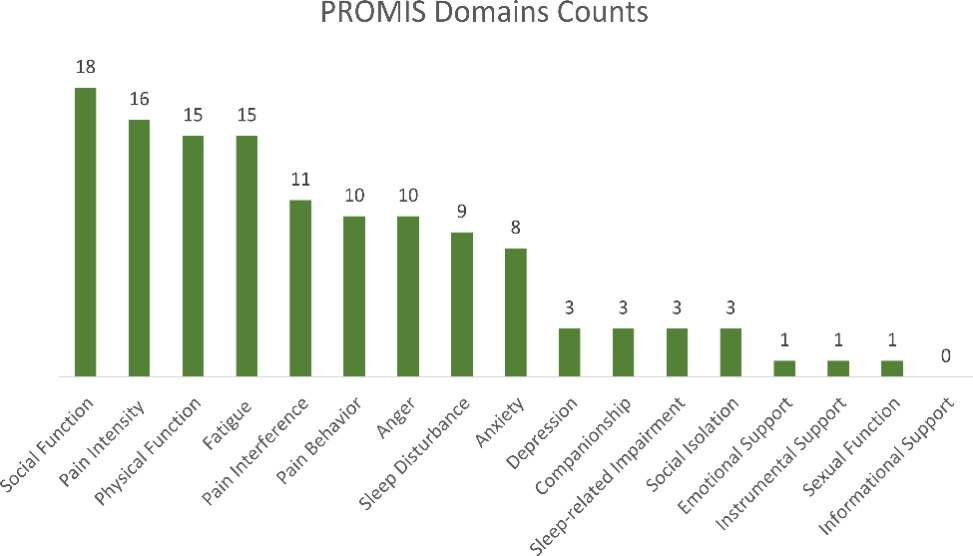
Number of times PROMIS-domains were selected by 26 participants

Figure 1 presents the most important PROMIS HRQOL domains for LBP selected by the participants. Out of 18 domains, participants selected 17 domains. The only domain that was not selected was “informational support”. The most frequently chosen domains were *social function, pain intensity, physical function, fatigue*, and *pain interference.* Table 4 presents the main themes and subthemes that emerged from the card sorting, and Appendix 3 presents the distribution of domain selection.

**Table 4:** The main themes and subthemes connected to the card sorting question derived from the participants’ statements during the selection of PROMIS HRQOL domains

Domain	Theme	Subtheme (# occurrence)
Pain Intensity	Description	Severe pain (9)
		Constant pain (5)
	Exacerbation/alleviation factors of pain	Being active increases the pain (3)
		Being in the same position for a long time increases the pain (1)
	Coping with pain	Changing body position decreases the pain (1)
		Living with and ignoring the pain (1)
	Effect/Consequence of having pain	Restriction in activities (12)
		Fatigue (3)
		Lack of sleep (3)
		Anger (2)
		Bad posture (1)
		Behavioral change (1)
		Thinking interference (1)
		Fear (1)
		Sadness (1)
		Surprise (1)
	Autonomy	Need help (1)
Social Function	Description	Restriction in social activity (7)
		Participating in social activities is difficult (3)
		Loss of pleasure (3)
	Factors leading to limitation of social activities	Pain restricts social activities (10)
		Anxiety restricts social activities (1)
		Lack of sleep restricts social activities (1)
	Coping with limitation of social activities	Need a wheelchair (1)
	Effect/Consequence of non-/performing social activities	Bother (2)
		Depression (2)
		Social participation exacerbates pain (2)
	Autonomy	Need help (1)
Physical Function	Description	Restriction in physical activity (3)
		Core (main) part in life (1)
		Muscle strength loss (1)
	Factors leading to limitation of physical function	Pain restricts general, social, physical, sport, housework, and work performance (life tasks) (9)
		Fear of pain restricts physical activity (2)
		Lack of sleep restricts physical activities (1)
	Coping	Avoid performing activities (1)
	Effect/Consequence of non-/performing physical activity	Performing activities exacerbates pain (1)
		Inability to perform activity makes me bothered (1)
		Inability to perform activity leads to frustration (1)
Fatigue	Description	Feeling tired (3)
	Factors leading to fatigue	Pain increases fatigue (5)
		Lack of sleep increases fatigue (4)
		Ability to perform work decreases fatigue (2)
	Coping with fatigue	Resting decreases fatigue (1)
	Effect/Consequence of fatigue	Restriction in activities/work (2)
		Depression (2)
		Anger (1)
		Pain exacerbation (1)
Pain Interference	Determinant	Family support decreases pain (1)
	Coping with pain	Living with and ignoring the pain (1)
	Effect/Consequence of pain interference	Restriction in general/physical/sport/housework/work activities (11)
		Restriction in social activities (6)
		Anger (1)
		Bother (1)
		Activity limitation is difficult (1)
	Description	Having pain makes patient angry (6)
		Having pain makes patient surprised (1)
		Having pain makes patient anxious (1)
		Having pain makes patient shout (1)
		Having pain makes patient talk about pain (1)
		Having pain makes patient physically limited (1)
Anger	Description	Anger is a handicap (1)
	Factors leading to anger	Pain causes anger (7)
		Inability to do daily activities causes anger (2)
		Vague condition (LBP) causes anger (1)
	Coping with anger	Workout – Being active (1)
	Effect/Consequence of anger	Mood changes (2)
		Discouraged/Frustration (2)
		Affects other HRQOL domains (1)
Sleep Disturbance	Description	Having sleep difficulty (4)
		Need to sleep (1)
	Factors leading to sleep problems	Pain interferes with sleep (4)
		Pain medication interferes with sleep (1)
		Inactive life style disturbs sleep (1)
	Effect/consequence of having sleep disturbance	Lack of concentration (2)
		Fatigue (2)
		Restriction in activity (2)
		Anger (1)
Anxiety	Description	Feeling of anxiety (2)
	Factors leading to anxiety	Pain causes anxiety (2)
		Lack of sleep causes anxiety (2)
		Lack of emotional support causes anxiety (1)
		Fearing of being physically limited causes anxiety (1)
	Effect/consequence of having anxiety	Social isolation (1)
	Coping with anxiety	Being active decreases anxiety (1)
Depression	Factors leading to depression	Pain impairs emotional status (2)
	Effect/consequence of having depression	Affects other HRQOL domains (1)
Companionship	Description	Lack of emotional support (1)
		Important part of social life (1)
	Factors leading to companionship problems	Having pain restricts social activities (3)
		Anger deteriorates social life (1)
		Not satisfied with sexual function (1)
Sleep-related impairment	Description	Uncomfortable with sleep because of pain (1)
	Effect/consequence of sleep problem	Restriction in activity (2)
		Increases pain intensity (1)
		Lack of pleasure (1)
		Memory problems (1)
		Lack of orientation (1)
		Affects other HRQOL domains (1)
Social Isolation	Factors leading to social isolation	Pain causes social isolation (2)
Emotional Support	Description	Lack of emotional support (2)
	Effect/consequence of emotional support	Emotional support decreases pain (1)
Instrumental Support	Description	Lack of instrumental support (1)
	Effect/consequence of lack of instrumental support	Lack of instrumental support leads to restriction in activities (1)
Sexual Function	Factors leading to sexual function problems	Pain restricts sexual activity (1)
	Effect/consequence of sexual function problems	Loss of joy (1)

### Social function

Eighteen participants selected the social function domain. Thirty-three statements were made by participants to elaborate on the importance of the social function domain with regard to LBP, forming five themes: *description, factors leading to limitations to social activities, coping with limitations to social activities, effect/consequence of not performing social activities*, and *autonomy*. Most participants indicated that they experienced difficulty and restriction in social activities with family and friends. Three participants stated that not performing social activities led to a lack of pleasure. Additionally, depression was noted by two participants as a result of restriction in social activities.

*“Your relation to social roles to interact with other people is affected by pain and anxiety, and you cannot focus on what you are doing”.* P15

*“So, yes, this my low back pain affects the type of activities that I want to do, and then yes … I feel that … it is impacting a little bit my enjoyment of life, whether I want to go, local journey, or travel where I know I am going to be in more pain, or doing activities where I could be in more pain”* P7

*“J’aime les… ce qu’on appelle les activités sociales. Alors, quand je peux pas faire ça, évidemment, ça me déprime et ça me… même si j’ai un bon moral, je veux dire, un moment donné on perd le goût. [I like the.. what we call social activities. So when I can’t do that, obviously, it depresses me and it makes me...even though I have a good spirit, I mean at some point you lose interest]”. P3*

### Pain intensity

Sixteen participants selected the pain intensity domain. Forty-eight statements were used by the participants to describe pain intensity in LBP, forming five themes: *description of pain, exacerbation/alleviation factors of pain, coping with pain, effect/consequence of having pain*, and *autonomy*. Pain intensity is important in LBP because of its severity and its contribution to restrictions in life tasks. The participants described pain as severe and constant. Most of the participants’ statements regarding pain intensity were related to the effect/consequence of having severe pain and its restriction on life tasks, fatigue, anxiety, fear, and lack of sleep. Regarding the factors that exacerbate/alleviate pain, three participants stated that performing regular activities increased the pain intensity, and one participant stated that being in the same position for a long time increased pain.

*“Pain intensity, because sometimes is better than other days. Mostly I have bad days. So I would say pain intensity could be so bad.”* P6.

*“I do a little bit work. If I do much the pain gets worse”* P21.

### Physical function

Fifteen participants selected the physical function domain. Twenty-one statements were made by participants to describe physical function in LBP, forming four themes: *description, factors leading to physical function limitation, coping*, and *effect/consequence of not performing physical activity*. Participants stated that physical function was an important factor in their LBP condition because of the restriction in activities, especially physical activities. Additionally, the participants stated that being physically limited made them anxious, fearful, and frustrated. One participant stated that physical function is an important domain because it is the core domain in life.

*“This is the one (*i.e., *physical function) affects me a lot because I have been always very physically”P21*

*“When I have an episode, I could be off, I do not do any activities, any physical activities for easily a month, a month and half. After that, I have to slowly come back, I couldn’t be myself or I could not go on my rhythm”.* P22

*“Donc, la fonction physique, c’est ce qui cause la douleur et en contre-partie. je suis frustré parce que je peux pas la faire comme il faut. [So, physical function, this is what causes the pain, and in return. I’m frustrated because I can’t do it right]”.* P5

### Fatigue

Fourteen participants selected the fatigue domain. Twenty-one statements were made by participants to describe fatigue, forming four themes: *description, factors leading to having fatigue, coping with fatigue, and effect/consequence of having fatigue*. Most of the participants’ statements were related to the exacerbation and alleviation factors of fatigue. Five participants stated that pain made them tired, three participants indicated that lack of sleep exacerbated fatigue, and two participants mentioned that being physically fit decreased the risk of fatigue.

*“The fatigue is because of back pain. It’s quicker, it’s a faster. My fatigue comes quicker because of my LBP. You know what I mean, like if I do not have a lower back pain I will not be as tired”.* P17

*“C’est sûr que je passe mon temps à me forcer… à avancer… puis là, un moment donné, c’est sûr que je viens fatiguée, faut que je m’assois. [Of course I spend my time forcing myself... to move forward... then, at some point, I become tired, I have to sit down].* P25

### Pain interference

Eleven participants selected the pain interference domain. Twenty-two statements were related to this domain, forming three themes: *determinants of pain, coping with pain*, and *effect/consequence of pain interference*. Participants stated that LBP mainly interfered with their physical and social tasks: general, physical, sports, housework, and work activities.

*“It [LBP] affects my ability to do tasks well. It might affect my interaction with colleagues. I think it interferes with many activities I have to do”. P7*

*“Et l’interférence de la douleur, je l’associe tout de suite, c’est la cause qui m’empêche. C’est la raison pour laquelle je ne peux pas faire les autres choses. [The pain interference, I associate it immediately, it is the cause that prevents me. This is the reason why I cannot do other things]”.* P5

### Integration of open-ended question and card-sorting data

In this study, the quantitative results were used to identify the most important domains according to the patients’ perspectives. The most frequently chosen domains in card sorting (quantitative part) were *social function, pain intensity, physical function, fatigue*, and *pain interference*. This was supported and explained by the results of the open-ended question, where the most frequently affected areas identified were pain description corresponding to pain severity, general physical activity restriction corresponding to physical function, and social activity and work performance restriction corresponding to social function.

## Discussion

This study aimed to identify the most important domains of HRQOL from the perspective of individuals with LBP. The PROMIS framework divides health into three categories: physical, social and mental health. In this study, physical health emerged as an important area to be considered from the spontaneous statements (open-ended questions) that participants expressed when they were asked about the effect of LBP on their quality of life. After physical health, patients nominated social and mental health as important. Similarly, the PROMIS card sorting method resulted in four out of the five most important domains pertaining to physical health: *pain intensity, physical function, fatigue*, and *pain interference*. The fifth most important domain pertained to social health, *social function*.

As part of physical health, *pain intensity, physical function* and *fatigue* were identified as the most important domains. Previous studies have shown that pain is considered the main symptom of LBP [38–40]. A longitudinal study showed that fatigue was frequently reported in people with chronic LBP [41]. Most participants in the current study reported that they used to do many activities before having LBP, but their LBP condition now restricted them. This is consistent with the WHO report on the burden of LBP that showed that LBP is a leading cause of disability in developed countries [40].

*“Social function”* was the most frequently selected domain. Social function was merged from two PROMIS domains: “*ability to participate in social roles and activities*” and “*satisfaction with social roles and activities*”. These two domains were combined because participants had difficulty differentiating between them. A systematic review showed that the social component of life was important for individuals with LBP [42]. In addition, the WHO reported that LBP affects work performance among individuals with LBP and is considered a leading cause of work absence and loss [40].

Findings from the PROMIS card sorting and the open-ended question showed that participants perceived physical health as most important compared to mental health. In the open-ended question, anxiety and depression were stated only twice, while during the PROMIS card sorting, these two domains were selected by 8 (31%) and 3 (11%) participants, respectively. A systematic review of the impact of LBP showed that anxiety and depression were frequently reported by individuals with LBP [42]. The findings of the current study may explain why our participants did not consider depression and anxiety to be important domains; these two domains were frequently stated as consequences of pain, fatigue, and restriction in physical and social activities. Therefore, participants might think that if the latter domains (i.e., pain, fatigue, and restriction in activities) were addressed, anxiety and depression may be reduced.

The overlap between the open-ended question and the PROMIS card sorting showed consistency between the two approaches. Both components showed that pain intensity and restriction in physical and social activities were perceived as important areas in LBP. However, fatigue was not frequently stated in the open-ended question statements, while it was perceived as important in the PROMIS card sorting. Participants expressed the general effect of LBP during the open-ended question reporting, while they indicated more specific domains when they were cued by the PROMIS card selection.

In a previous study, Zidarov et al. (2020) identified the most important HRQOL domains among individuals with chronic pain: *pain interference, pain intensity, physical function, sleep disturbance, anxiety, depression, ability to participate in social roles and activities, fatigue, sleep-related impairments and self-efficacy* [43]. These domains can be mapped to the physical health, social health and mental health domains of the PROMIS. All these domains, except mental health, were also considered the most important domains for LBP in the current study. According to LBP clinicians and experts, the NIH identifies the minimal dataset that is recommended to describe people with LBP: pain intensity, pain interference, physical function, depression, sleep disturbance, and catastrophizing [22]. Three of the domains identified by the NIH were also identified by participants in the current study: *pain intensity, pain interference* and *physical function. Sleep disturbance*, based on frequency, was not selected as an important domain by participants in the current study. However, nine participants selected sleep disturbance and three selected sleep-related impairment as important domains in this study. Catastrophizing was not included in the PROMIS framework. However, the literature shows that catastrophizing is used to refer to anxiety disorder [44], which was also not selected frequently by participants in this study. Participants in this study believed that anxiety and depression were not directly caused by LBP but rather were a result of pain, fatigue, and activity limitations. The results from this study provide guidance on which domains can be systematically collected in clinical care for LBP. Other relevant domains for a specific context may be selected by clinical teams or individuals with LBP.

Several tools are used to measure health outcomes among people with LBP, such as the Short-Form 36 (SF-36) [45] and WHO Quality of Life-BREF (WHO-QOL-BREF) [46], which are frequently used HRQOL patient reported outcome measures in LBP. Both tools require approximately 16 to 55 min to complete, and they do not assess all of the HRQOL domains that are perceived as important by LBP participants. Neither measure assesses the pain intensity domain. Another approach, guided by information from this study on the most important domains for individuals with LBP, is to select items from item banks that are most informative to measure each domain. This can be achieved using a measurement system such as PROMIS computerized adaptive testing to efficiently assess the selected domains and decrease the response burden.

### Limitations

There are some limitations of this study that need to be noted. Two domains of PROMIS HRQOL that could be considered important for the study were not presented to patients during the interview: self-efficacy and cognitive function. These domains were not nominated by participants in response to the open-ended question. Additionally, the findings of this study cannot be generalized to all people with LBP because the participating people were from one province in Canada and a specific health context.

## Conclusion

The most important domains of HRQOL perceived by participants were pain intensity, social function, physical function, fatigue, and pain interference. Identifying the most important domains of HRQOL may help clinicians focus on and target these areas during the development of treatment plans. In turn, by targeting interventions to address limitations in these domains, this may improve the health status of people with LBP.

## Electronic supplementary material

Below is the link to the electronic supplementary material.

Supplementary Material 1

## Data Availability

The datasets generated and/or analyzed during the current study are not publicly available due to ethical considerations but are available from the corresponding author on reasonable request.

## List of abbreviations

HRQOL: Health-Related Quality of Life
ICF: International Classification of Functioning, Disability and Health
IRT: Item Response Theory
LBP: Low Back Pain
NIH: National Institute of Health
PROMIS: Patient-Reported Outcomes Measurement Information System
WHO: World Health Organization
WHO-QOL-BREF: WHO Quality of Life-BREF
