# Supplementary material for: Identifying domains of health-related quality of life: the perspective of individuals with low back pain
Source: J Patient Rep Outcomes. 2023 Jul 26;7:79. doi: 10.1186/s41687-023-00597-5 (PMC10371923; doi:10.1186/s41687-023-00597-5)
Supplement: Supplementary file 1 — Supplementary Material 1 [file 41687_2023_597_MOESM1_ESM.docx]

## **Appendices**

**Appendix 1: Interviews with CLBP individuals about health-related quality of life**

**Name interviewer:**

**Name respondent:**

**Date:**

**Time:**

**Place:**

1. **Introduction**

Introducing myself

My name is………, and I am a student at McGill working with Dr. Sara Ahmed. We are conducting a research project on health-related quality of life in LBP.

Subject

Health-related quality of life comprises of different domains. Our research aims to assess, by means of interviews, which of these domains are the most relevant in LBP, from the individuals with LBP perspective. In this way, a multidimensional questionnaire may be developed to assess comprehensively health-related quality of life in individuals with LBP.

Confidentiality

The research results will be used for scientific purposes only. Your personal information will not be identifiable.

Duration

The interview will take 15-20 minutes.

Audio recording

During the interview I will take notes. In addition, I would like to audio record the interview, as it will improve the accuracy of my report. Please, be assured that the audio recordings will be deleted when the research has been completed.

Do you object to the use of an audio recorder during the interview? YES/NO

Any questions or remarks at this stage?

1. **Central focus**

We will start the interview. Remember that there are no right or wrong answers. It is about your personal situation and your personal experience. Take your time before answering any question. Let me know if you need a break.

- 1. Questions related to LPB
- Since when have you had LBP?
- Since when have you received treatment for your LBP?
- Have you got other illnesses or complaints besides LBP? YES/NO

2.2 Domains

*2.2.1 Open question*

We now have come to the part that deals with the different domains of quality of life. I would like to begin with the following question:

- How does LBP affect your quality of life?
  - 1. *Cards with domains*

I will place eighteen cards in front of you. On these cards, nineteen domains of quality of life are described, each accompanied by a few examples. I would like you to choose five domains of quality of life, which you feel are the most affected by your LBP. Try not to focus on the example questions, as you don’t need to answer them. They only indicate what the theme signifies. Concentrate instead on the general contents of each domain.

You indicate that the following five domains are affected the most by your LBP:

Carefully study these cards again. Arrange the cards from most important to least important.

The way you have arranged the cards indicates that the following domains are the most relevant in connection with your LBP:


- Can you indicate why this domain is important with regard to your LBP?


- Do you have any additional comments?
- This is the end of the interview. Do you have any questions or remarks?

Thank you for your time and cooperation.

**Appendix 2: PROMIS HRQL Domains** [30]

| Domain | Description |
| --- | --- |
| Ability to Participate in Social Roles and Activities | “Assesses the perceived ability to perform one’s usual social roles and activities (e.g., “I have to limit my regular family activities”)”  *e.g.: Do you have to limit the things you do for fun with others?* |
| Anger | “Assesses angry mood (e.g., irritability, frustration), negative social cognitions (e.g., interpersonal sensitivity, envy, and disagreeableness), verbal aggression, and efforts to control anger. Anger is distinguished by attitudes of hostility and cynicism and is often associated with experiences of frustration impeding goal-directed behavior”.  *e.g.: I was irritated more than people knew* |
| Anxiety | “Assesses fear (e.g., fearfulness, feelings of panic), anxious misery (e.g., worry, dread), hyperarousal (e.g., tension, nervousness, restlessness), and somatic symptoms related to arousal (e.g., racing or pounding heart, dizziness)”.  *e.g.: I felt anxious* |
| Companionship | “Assesses perceived availability of someone with whom to share enjoyable social activities such as visiting, talking, celebrations, etc”  *e.g.: Do you have someone with whom to relax?* |
| Depression | “Assesses negative mood (e.g., sadness, guilt), negative views of the self (e.g., self-criticism, worthlessness), negative social cognition (e.g., loneliness, interpersonal alienation), and decreased positive affect and engagement (e.g., loss of interest, loss of meaning and purpose)”.  *e.g.: I felt upset for no reason* |
| Emotional Support | “assesses perceived feelings of being cared for and valued as a person; having confidant relationships”  *e.g.: Do you have someone to confide in or talk to about yourself or your problems?* |
| Fatigue | “Assesses fatigue from mild subjective feelings of tiredness to an overwhelming, debilitating, and sustained sense of exhaustion that is likely to decrease one’s ability to carry out daily activities, including the ability to work effectively and to function at one’s usual level in family or social roles”.  *e.g.: How often did you have to push yourself to get things done because of your fatigue?* |
| Informational Support | “Assesses perceived feelings of being cared for and valued as a person; having confidant relationships”.  *e.g.: Do you have someone to turn to for suggestions about how to deal with a problem?* |
| Instrumental Support | “Assesses perceived availability of assistance with material, cognitive or task performance”  *e.g.: Do you have someone to take you to the doctor if you need it?* |
| Pain Behavior | “Assesses external manifestations of experiencing pain”  *e.g.: When I was in pain I screamed* |
| Pain Intensity | “Assesses how much a person hurts” |
| Pain Interference | “Assesses the consequences of pain on relevant aspects of persons’ lives and may include the impact of pain on social, cognitive, emotional, physical, and recreational activities as well as sleep and enjoyment in life”  *e.g.: How much did pain interfere with your close personal relationships?* |
| Physical Function | “Assesses one's ability to carry out activities that require physical actions, ranging from self-care (activities of daily living) to more complex activities that require a combination of skills, often within a social context”  *e.g.: Are you able to put on a pullover sweater?* |
| Satisfaction with Social Roles and Activities | “Assesses satisfaction with performing one’s usual social roles and activities (e.g., “I am satisfied with my ability to participate in family activities”)”  *e.g.: Are you satisfied with your ability to do household chores/tasks?* |
| Sexual Function | “Mental and physical functions related to the sexual act, including the arousal, preparatory, orgasmic and resolution stages” |
| Sleep disturbance | “Assesses perceptions of sleep quality, sleep depth, and restoration associated with sleep; perceived difficulties and concerns with getting to sleep or staying asleep; and perceptions of the adequacy of and satisfaction with sleep”  *e.g.: I tossed and turned at night* |
| Sleep-Related Impairment | “Assesses perceptions of alertness, sleepiness, and tiredness during usual waking hours, and the perceived functional impairments during wakefulness associated with sleep problems or impaired alertness”  *e.g.: I had a hard time getting things done because I was sleepy* |
| Social Isolation | “Assesses perceptions of being avoided, excluded, detached, disconnected from, or unknown by, others.”  *e.g.: Do you feel that people avoid talking to you?* |

**Appendix 3: Important Selected Domain**

| Domain | 1^st^ important selected domain (N) | 2^nd^ important selected domain (N) | 3^rd^ important selected domain (N) | 4^th^ important selected domain (N) | 5^th^ important selected domain (N) | 6^th^ important selected domain (N) | 7^th^ important selected domain (N) | 8^th^ important selected domain (N) | 9^th^ important selected domain (N) |
| --- | --- | --- | --- | --- | --- | --- | --- | --- | --- |
| Pain Intensity | 6 | 4 | 3 | 3 | 0 | 0 | 0 | 0 | 0 |
| Physical Function | 5 | 4 | 1 | 4 | 1 | 0 | 0 | 0 | 0 |
| Pain Interference | 4 | 1 | 1 | 2 | 1 | 2 | 0 | 0 | 0 |
| Sleep disturbance | 4 | 1 | 3 | 0 | 0 | 0 | 0 | 1 | 0 |
| Social Function | 3 | 4 | 2 | 2 | 4 | 2 | 0 | 0 | 1 |
| Anger | 1 | 1 | 1 | 2 | 4 | 0 | 1 | 0 | 0 |
| Anxiety | 1 | 0 | 1 | 2 | 1 | 3 | 0 | 0 | 0 |
| Pain Behavior | 1 | 2 | 3 | 2 | 3 | 0 | 0 | 0 | 0 |
| Companionship | 0 | 0 | 1 | 0 | 2 | 0 | 0 | 0 | 0 |
| Depression | 0 | 0 | 1 | 1 | 0 | 1 | 0 | 0 | 0 |
| Emotional Support | 0 | 0 | 0 | 0 | 1 | 0 | 0 | 0 | 0 |
| Fatigue | 0 | 4 | 6 | 3 | 2 | 0 | 0 | 0 | 0 |
| Informational Support | 0 | 0 | 0 | 0 | 0 | 0 | 0 | 0 | 0 |
| Instrumental Support | 0 | 0 | 0 | 0 | 1 | 0 | 0 | 0 | 0 |
| Sexual Function | 0 | 0 | 1 | 0 | 0 | 0 | 0 | 0 | 0 |
| Sleep-Related Impairment | 0 | 3 | 0 | 0 | 0 | 0 | 0 | 0 | 0 |
| Social Isolation | 0 | 0 | 0 | 1 | 1 | 1 | 0 | 0 | 0 |

N: number of participants
